# Treatment of bilateral popliteal artery and right anterior tibial artery aneurysm with endovascular stent grafting

**DOI:** 10.1186/1749-8090-8-S1-P88

**Published:** 2013-09-11

**Authors:** O Tetik, Y Besir, S Surer, O Rodoplu, M Melek

**Affiliations:** 1 Department of Cardiovascular Surgery, Manisa Celal Bayar University School of Medicine, Manisa, Turkey; 2 Department of Cardiovascular Surgery, Ataturk Training and Research Hospital, Izmir, Turkey; 3 Department of Cardiovascular Surgery, Yuksek Ihtisas Training and Research Hospital, Bursa, Turkey

## Background

Although rare, popliteal artery aneurysms form the majority of peripheral arterial aneurysms and are generally asymptomatic. They usually cause embolic events such as extremity ischemia. In this paper, we present a patient successfully treated with endovascular stent grafting for bilateral popliteal artery aneurysm.

## Methods

A 67-year-old woman presented to our clinic with pain at the back of bilateral knee joints. Physical examination showed manually palpable distal arterial pulses and a pulsatile mass at bilateral popliteal fossae. Arterial Doppler ultrasonography revealed bilateral popliteal arterial aneurysms. Multislice computerized tomography showed an aneurysm starting from distal femoral artery and involving entire popliteal artery at the left side and aneurysmal dilatation in both popliteal artery and proximal segment of the anterior tibial artery at the right side. Screening tests failed to show any additional aneurysms at other sites.

## Results

Both popliteal artery and right tibial artery aneurysms were successfully treated with stent grafts at one month interval. The patient was discharged with full recovery after an uneventful postoperative course.

## Conclusions

Management of popliteal artery aneurysms are very important because the complications threaten both limb and life.

